# Early symptoms and sensations as predictors of lung cancer: a machine learning multivariate model

**DOI:** 10.1038/s41598-019-52915-x

**Published:** 2019-11-11

**Authors:** Adrian Levitsky, Maria Pernemalm, Britt-Marie Bernhardson, Jenny Forshed, Karl Kölbeck, Maria Olin, Roger Henriksson, Janne Lehtiö, Carol Tishelman, Lars E. Eriksson

**Affiliations:** 1 Division of Innovative Care Research, Department of Learning, Informatics, Management and Ethics (LIME), Karolinska Institutet, SE-171 77 Solna, Sweden; 2 Cancer Proteomics Mass Spectrometry, Department of Oncology-Pathology, Karolinska Institutet, Science for Life Laboratory, SE-171 65 Solna, Sweden; 3 Lung Oncology Center, Cancer Theme, Karolinska University Hospital, SE-171 76 Solna, Sweden; 4 Department of Radiation Sciences and Oncology, University of Umeå, SE-901 87 Umeå, Sweden; 5 Center for Health Economy, Informatics and Health System Research (CHIS), Stockholm Health Care Services (SLSO), Region Stockholm, SE-113 65 Stockholm, Sweden; 6 The Centre for Rural Medicine (Glesbygdsmedicinskt Centrum GMC), Region Västerbotten, SE-923 31 Storuman, Sweden; 7 School of Health Sciences, City, University of London, Northampton Square, London, EC1V 0HB United Kingdom; 8 Department of Infectious Diseases, Karolinska University Hospital, SE-141 86 Huddinge, Sweden

**Keywords:** Lung cancer, Risk factors, Signs and symptoms, Respiratory signs and symptoms, Statistics

## Abstract

The aim of this study was to identify a combination of early predictive symptoms/sensations attributable to primary lung cancer (LC). An interactive e-questionnaire comprised of pre-diagnostic descriptors of first symptoms/sensations was administered to patients referred for suspected LC. Respondents were included in the present analysis only if they later received a primary LC diagnosis or had no cancer; and inclusion of each descriptor required ≥4 observations. Fully-completed data from 506/670 individuals later diagnosed with primary LC (n = 311) or no cancer (n = 195) were modelled with orthogonal projections to latent structures (OPLS). After analysing 145/285 descriptors, meeting inclusion criteria, through randomised seven-fold cross-validation (six-fold training set: n = 433; test set: n = 73), 63 provided best LC prediction. The most-significant LC-positive descriptors included a cough that varied over the day, back pain/aches/discomfort, early satiety, appetite loss, and having less strength. Upon combining the descriptors with the background variables current smoking, a cold/flu or pneumonia within the past two years, female sex, older age, a history of COPD (positive LC-association); antibiotics within the past two years, and a history of pneumonia (negative LC-association); the resulting 70-variable model had accurate cross-validated test set performance: area under the ROC curve = 0.767 (descriptors only: 0.736/background predictors only: 0.652), sensitivity = 84.8% (73.9/76.1%, respectively), specificity = 55.6% (66.7/51.9%, respectively). In conclusion, accurate prediction of LC was found through 63 early symptoms/sensations and seven background factors. Further research and precision in this model may lead to a tool for referral and LC diagnostic decision-making.

## Introduction

Lung cancer (LC) remains the leading cause of cancer-related mortality^1–3^. While LC generally manifests with early symptoms and sensations, they are often so diffuse that care-seeking may be delayed^4,5^. Traditional risk factors, i.e. smoking, are not optimal in discriminating LC due to poor model performance^6,7^, thus, keen general practitioner vigilance^8–10^ and quick access to sensitive screening tools are needed^10–12^. While low-dose computerised tomography has been shown to be an important screening tool for LC^13,14^, it also suffers a high false-positive rate^13–15^ and should only be applied for particular risk groups. Thus, the need to identify early risk symptoms and sensations of LC that can flag individuals for screening and early detection remains^9,10^; this can be achieved from in-depth early symptomatic investigations.

Earlier identification of LC symptoms and sensations would have a major impact on overall LC mortality due to profoundly greater survival in early-identified stages^16^. Large cohort investigations from diffuse general practice medical records have thus far uncovered some LC-risk signs and symptoms, e.g. haemoptysis, dyspnoea, chest pain, cough, appetite loss and/or weight loss up to two years before diagnosis^17–20^. Only one prospective study^21^, to our knowledge, evaluated a symptom survey administered to patients referred for LC investigation before the individuals met a specialist or had received any primary LC diagnosis. Haemoptysis was a possible LC predictor, although only twenty descriptors were investigated^21^. A driving need thus remains for identifying a combination of pre-diagnostic individual descriptors that can predict primary LC.

### Study aim

This study was conducted to fill the gap left by limited investigations of patient-reported pre-diagnostic LC descriptors, contributing a more thorough investigation of patient experiences. The aim of this study is thus to identify a combination of early predictive symptoms and sensations attributable to LC.

## Methods

### Study conduction and sample

After approval by the Stockholm regional ethics board (EPN: ref no 2014/1290–32), data was collected from September 2014–November 2015. In Stockholm County, diagnostic work-up for suspected LC is centralised to Karolinska University Hospital (KUH). Thus, all consecutive patients referred to KUH were asked to participate in the study and sent written study information before their first scheduled visit. Upon the first visit, written informed consent was obtained. Patients then completed the Patient EXperience of Bodily Changes for Lung Cancer Investigation (PEX-LC) e-questionnaire on a touch screen user interface on a smart tablet directly before their clinical visit with a pulmonary medicine physician. Research assistants were available for help. Medical records of eventual diagnosis were later retrieved, with a follow-up of at least one year after questionnaire completion. This study was carried out according to the Declaration of Helsinki and data were anonymized to protect the privacy of the study participants.

### The PEX-LC instrument

The PEX-LC instrument is an e-questionnaire focusing on patients’ own specific pre-diagnostic descriptions of early symptoms or sensations, hereafter referred to as descriptors. The instrument was derived from prior qualitative interviews (n = 60) conducted at several Swedish lung medicine departments. PEX-LC consists of 11 individualised, interactive modules on a touch screen smart tablet: Background (e.g. sociodemographic characteristics, comorbidities and smoking habits), Breathing Difficulties, Cough, Phlegm/Expectorates, Pain/Aches/Discomfort, Fatigue, Voice Changes, Appetite/Eating/Taste Changes, Olfactory Changes, Fever/Chills/Sweating, and Other Changes (e.g. general physical condition, malaise, or other emotional changes). There are 342 potential items; 285 descriptors indicative of the first symptoms/sensations the patient noticed that had caused a change in their lives, and 57 background variables. Patient-reported recall of early descriptors is recorded in binary form (“yes”/”no”). PEX-LC was tailored to allow each individual participant to complete only those items appropriate for the specific individual’s onset of symptoms or sensations.

### Statistical analyses

Descriptors and background variables meeting inclusion criteria (≥4 observations for LC and for no cancer (NC), respectively (software default, SIMCA v.14.1)) were first analysed by principal component analysis (PCA) for data inspection for potential biases in the data, such as clusters or outliers which could skew findings^22^. Orthogonal projections to latent structures (OPLS) discriminant analysis (detailed description below) with cross-validation (CV) was then carried out to class-separate the data between the predicted (LC vs. NC) and orthogonal (structured noise) states^23–26^ (SIMCA v.14.1). Univariate associations to LC were analysed with binary logistic regression, and proportional (e.g. gender) and continuous data (age) were analysed with Pearson’s chi-squared tests and Independent Samples Mann-Whitney U tests, respectively (IBM SPSS v.24).

### Orthogonal projections to latent structures (OPLS) discriminant analysis

An OPLS modelling approach was utilised to analyse variables (descriptors) covarying with outcome (LC or NC)^23–26^. Analyses were performed with SIMCA v.14.1, Umetrics™ Suite, Sartorius Stedim Biotech. Inclusion criteria were full-module completion (no missing data) and ≥4 observations for descriptors, and a diagnosis of primary LC or NC (other cancer diagnoses led to exclusion) for patients.

Cross-validation estimates the predictive performance of a model, thus ensuring model reliability. Applying CV with OPLS in SIMCA avoids model overfitting by only retaining significant components in the model^27^. *K*-fold CV was carried out with 1/7^th^ of the dataset being excluded for each round (software default^28^) up until and including the sixth group (six-fold CV for the training set). The seventh group was the CV test set, independent of model training.

**Figure 1:**
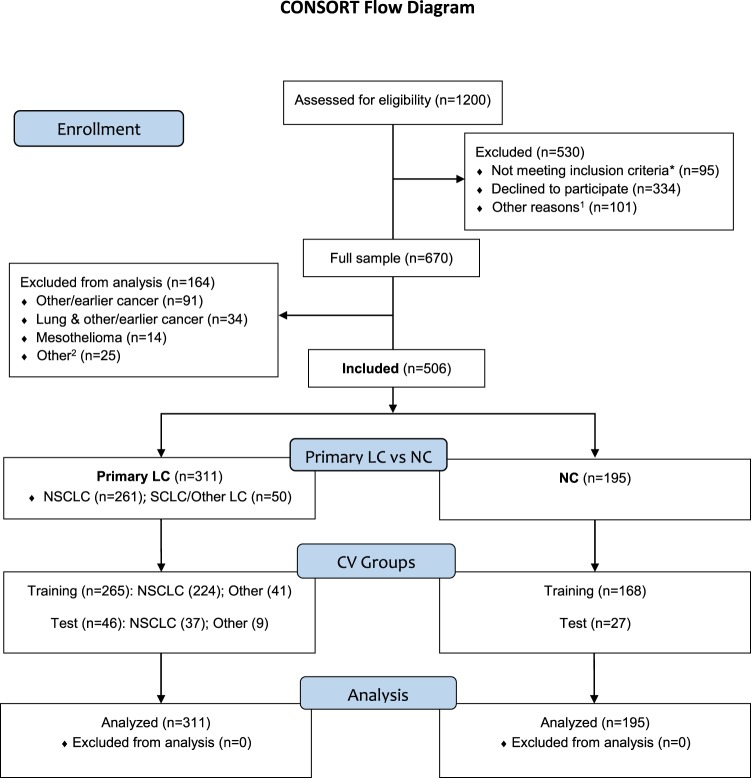
CONSORT flow diagram: The PEX-LC lung cancer investigation cohort. This figure is based on the CONSORT 2010 flow diagram. As this was not a randomised intervention trial, it has been modified to suit this cohort study accordingly. Primary LC: primary lung cancer (no other cancer); NC, no cancer; NSCLC: non-small cell lung cancer (adenocarcinoma, n = 200; squamous cell carcinoma, n = 45; not otherwise specified (NOS), n = 5; other NSCLC (adenosquamous lung carcinoma (n = 4), large cell neuroendocrine carcinoma (n = 3); large cell carcinoma, adenoid cystic carcinoma of the lung, adenoid carcinoma with neuroendocrine differentiation, and mucoepidermoid carcinoma of the lung (n = 1, respectively)); SCLC: Small cell lung cancer (includes one individual with combined SCLC) (n = 24); Other LC: carcinoid, n = 9; no histology, n = 17. *Not meeting inclusion criteria: translator required (n = 50), consent withdrawn/missing (n = 15); missing data (n = 5); other reason such as or pain, illness, or other medical condition (n = 25). ¹ Other reasons: Limited time of the visit or lack of resources (staff) at the clinic (n = 47); hospitalisations (n = 34); deaths (n = 20). ² Other: Medical records non-consent (n = 4); unconfirmed, possible lung cancer (n = 3); undiagnosed cancer (n = 2); death before clinical investigation (n = 1); participant withdrew clinical investigation (n = 2); previous lung cancer (n = 1); incomplete modules (n = 12). Primary LC: Current/previous comorbidities include Crohn’s disease, diabetes, gout, lymphedema, pulmonary fibrosis, fibromyalgia, sarcoidosis (n = 1, respectively); rheumatoid arthritis (n = 2); asbestos-related disease (n = 3); heart disease or anaemia (n = 4, respectively); chronic bronchitis (n = 5); angina pectoris (n = 15); emphysema (n = 17); pulmonary oedema (n = 33); asthma (n = 35); chronic obstructive lung disease (COPD, n = 70); pneumonia (n = 73); no comorbidities/unknown (n = 113). NC (no malignant cancer): Diagnoses included Castleman’s disease, empyema, systemic lupus erythematosus, gout, polymyositis, previous granulomatosis with polyangiitis, haemoptysis, tuberculosis (n = 1, respectively); benign hamartoma, resected benign hamartoma, tularaemia (n = 2, respectively); diabetes, sarcoidosis (n = 3, respectively). Current/previous conditions, NC: asbestos-related disease, bronchitis, kidney failure or lung embolism (n = 1, respectively); anaemia (n = 3); chronic bronchitis (n = 5); emphysema (n = 6); angina pectoris (n = 7); pulmonary oedema (n = 25); heart disease or COPD (n = 26, respectively); asthma (n = 34); pneumonia (n = 58); no diagnosis/unknown (n = 73).

To ensure cohort representativeness and to remove any potential bias created by chance due to row placement^27^, all seven CV groups were created by block-randomisation to have similar proportions of LC (~60%) vs. NC (~40%) as expressed in the entire dataset, in addition to randomised row placement. This block-randomisation also took full dataset representativeness of LC histology (Fig. 1) into consideration (non-small cell, 80–85% vs. small cell/other, 15–20% for each of the seven groups).

### Model selection

Multivariate regression models through OPLS were created through selection from key criteria, including PCA loadings for background variables, OPLS projection loadings, explained variance, and sensitivity over specificity, listed as follows. The first model included potential LC-associated background variables and descriptors meeting inclusion criteria, which served as the basis for all models as it projected all variables’ relative importance for overall model contribution. The theoretical foundation of PLS/OPLS is that it is hypothetically more precise with a higher load of potential variance-explaining variables from multi-dimensional interactions^28^. Variables were thus excluded sequentially through visual inspection of OPLS regression coefficients (which reflect each variable’s importance in relation to the first (predictive) component) as well as through inspection of variable importance for the projection (VIP) values (which indicate overall model contribution, both to prediction and to structured noise). Maximal explained variance of LC within the training set (R^2^) and CV-explained variance in the test set (Q^2^; >50%, respectively – considered good predictability^27^) was the criteria for a model to be evaluated, with highest possible R^2^ and Q^2^ values being prioritised. Thus, before each sequential variable would be totally removed from a model, explained LC variance (R^2^ and Q^2^) would be cross-referenced pre- and post-removal. Variables offering no model contribution were removed sequentially in this fashion. As the seven CV groups were always the same, to ensure that this sequential removal of variables did not overfit the model for the CV test set, 100 model simulations of randomised outcome (LC or NC) were carried out to ensure that by-chance R^2^ and Q^2^ were in all 100 instances worse than final model metrics.

The final model was chosen by selecting a cut-off with high sensitivity over specificity in the CV test set. Areas under the receiver operating characteristic (ROC) curves (AUC) for the CV test set were calculated from OPLS-generated LC prediction scores from each model, and were compared to find the most clinically-applicable model – with the maximal sensitivity over specificity ROC point by the Youden’s index – in IBM SPSS v.24. Acceptable model discrimination for the test set was determined by AUC > 0.7^29^.

## Results

Of the 1200 potentially-eligible patients investigated for suspected LC, 670 individuals agreed to participate (age and gender did not differ between those participating and the remaining potentially-eligible patients, data not shown). Of the participating patients, 506 were later diagnosed with primary LC or NC (n = 311, 195, respectively); the remaining 164 patients were excluded primarily due to different/multiple diagnoses (Fig. 1). The analysed sample was marginally, although statistically significantly younger, and more often current smokers than the excluded group (basic demographics, Table 1).

**Table 1:** Patient characteristics in the total PEX-LC cohort.

Variable	Analysed (n = 506)^a^	Excluded (n = 164)^a^	P value
Age, years (Median (IQR))	70 (63–75)	72 (64.3–78)	**0.008**
Sex, females	249 (49.2)	80 (48.8)	0.924
Current smokers*	148 (29.2)	28 (17.1)	**0.002**
Confirmed history of asthma	68 (13.4)	13 (7.9)	0.060
Confirmed history of COPD	93 (18.4)	20 (12.2)	0.066
Confirmed history of pneumonia	126 (24.9)	38 (23.2)	0.654
Antibiotics, past 2 years	193 (38.1)	52 (31.7)	0.137
Cold/flu/pneumonia, past 2 years	351 (69.4)	104 (63.4)	0.156

To compare patient characteristics between the individuals fulfilling study criteria (lung cancer or no cancer = analysed) and the remainder of the cohort (excluded), chi-squared tests (Fisher’s exact tests if expected counts < 5) were utilised to compare proportional data (e.g. proportion of females or current smokers), and Independent Samples Mann-Whitney U tests were utilised to compare continuous data (age).

^a^All variables are recorded in numbers (% proportions in parentheses), unless specified. *Current smokers includes individuals who recently quit smoking (within the past 1 year). IQR: interquartile range; COPD: chronic obstructive pulmonary disease. History of asthma, COPD or pneumonia, respectively, are physician-confirmed. Bolded two-sided p-values < 0.050 were considered statistically significant.

### PCA: Data inspection of included descriptors

**Figure 2:**
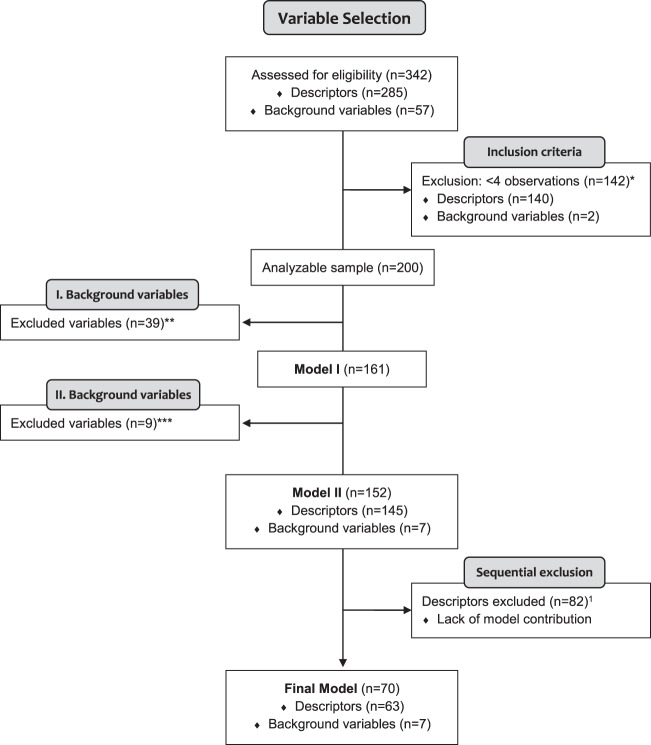
Variable selection flow diagram for the PEX-LC analysis. *The first exclusion step removed variables with limited observations (<4 observations of “yes” per variable for each outcome: lung cancer (LC) vs. no cancer). These variables are shown in Supplementary Table S1. **For step 1 of background variable removal for potentially-analysable results, the majority were not included due to lack of significant univariate associations to LC and/or were not previously-reported LC risk signs (n = 35/39). Ordinal smoking status (never-smokers, past smokers, current smokers), living alone, and university-level education were not included due to potentially overfitting the model, and weight loss was not included due to a large proportion of missing data. These variables are shown in S1 Table. ***For step 2 of background variable removal, the majority had principal component analysis loadings and orthogonal projections to latent structures variable importance for the projection (VIP) scores < 1 (n = 8). The past smokers (vs. non-smokers) variable was not included due to the potential risk of overfitting the model, as current smokers included those who quit smoking within the past 1 year. These variables are shown in Supplementary Table S2. ^1^Descriptors with minimal model contribution (Supplementary Table S2) were sequentially removed (n = 82) until maximal model performance could be achieved with 70 variables. The final model selection process including performance of additional models by variable count is shown in Supplementary Fig. S2A,B.

A PCA was performed on 145/285 early descriptors together with 16/57 background variables. The remaining variables were excluded due to not meeting inclusion criteria (<4 observations in LC or NC, respectively: 140 descriptors, two background variables), or, additionally, if they were background variables that either demonstrated no univariate associations to LC, would potentially overfit the model, or were not known LC risk factors (n = 39) (variable selection process, Model I: Fig. 2; excluded variables: Supplementary Table S1). In the next step, 9/16 background variables were removed due to lack of explained variance (PCA loadings <0.1) or overfitting the model (Model II: Fig. 2, excluded variables: Supplementary Table S2). Thus, the next and final PCA included seven background variables (Table 2). No irregular clustering or outliers were found among individuals with LC or NC (Supplementary Fig. S1). There were no differences in individual score distributions among the PCA quadrants when having inspected for variables such as age, smoking, sex, site of enrolment, LC histology or stage, and CV group (not shown).

**Table 2:** Identified descriptors and background factors for maximal lung cancer prediction performance.

BACKGROUND	
**Current smoking**	Confirmed history of COPD
**A cold, flu or pneumonia within the past 2 years**	Confirmed history of pneumonia*
**Female sex**	Antibiotics within the past 2 years*
**Older age (+1 SD, unit-variance scaled age)**	
**BREATHING**	
5: Wheezing/panting*	30: Breathing worse when I lay down*
7: Gasped for breath	31: Breathing worse due to high humidity
12: Felt thickness in throat	33: Breathing worse due to coldness*
21: Breathing sound: Whistled	35: Breathing worse during certain times of the day*
**29: Breathing worse upon exertion**	
**COUGH**	
3: Sudden, loud cough*	11: Needed to clear my throat*
4: Hacking cough*	**29: Cough varied over the day**
5: Wheezing cough*	35: Cough varied over the year
6: Irritating, dry cough	63: Cough occurred/worsened when I exerted myself*
7: Coughed until I lost my breath, choked and/or vomited*	64: Cough occurred/worsened when I breathed deeply*
8: Cough attacks*	68: Cough worsened by high humidity
10: Small coughs*	
**PHLEGM/EXPECTORATES**	
3: Decreased amount*	24: Thin, fluid-like consistency*
6: White mucus or sputum*	25: Taffy-like/viscous consistency*
**15: Haemoptysis/hematemesis (blood-mixed/brown sputum)**	
**PAIN/ACHES/DISCOMFORT**	
3: Hurting: Comes and goes	67: Heartburn
**8: Aches: Consistent**	201: Pain/aches/discomfort: Throat*
9: Aches: Comes and goes	204: Pain/aches/discomfort: Shoulder blade
10_11_12: Aches: Positional/breathing-based	207: Pain/aches/discomfort: Shoulder(s)
14: Pain: Consistent	210: Pain/aches/discomfort: Neck
16_17_18: Pain: Positional/breathing-based*	213: Pain/aches/discomfort: Chest
27: Cramping aches/pains: Comes and goes*	**223: Pain/aches/discomfort: Back**
39: Dull aches/pain: Comes and goes	227: Pain/aches/discomfort: Moves around*
49: Tenderness	
**FATIGUE**	**VOICE CHANGES**
**3: Less strength, got weaker**	1: Voice got more hoarse
4: Legs cannot cope	**2: Voice got more rough/coarse**
11: Felt constant tiredness, weakness, or lack of energy*	6: Cleared my throat more when I talked*
**APPETITE/EATING/TASTE CHANGES**	**OLFACTORY CHANGES**
**1: Appetite loss**	1: More difficult to distinguish smells
2: Enjoyed food less than before	2: Lost sense of smell*
**5: Early satiety (feeling full quicker)**	**3: Heightened sensitivity to different smells**
**FEVER**	**OTHER CHANGES**
1: Chills*	1: Cramps in calves
4: Felt cold	10: Drier skin*
13: Night sweats	13: Drier mouth
	19: Feeling unfit

Variables included in the final model (n = 70) are shown, including 7 background variables and 63 descriptors. Numbers indicate the identifiers of each of the included descriptors for each respective module and serve as a key to the regression coefficients shown in Supplementary Fig. S3. Of originally 285 descriptors, 145 met inclusion criteria (at least 4 observations in each group, lung cancer or no cancer). Additionally-excluded descriptors (n = 82) and background variables (n = 9) for model finalisation are indicated in Supplementary Table S2. History of chronic obstructive pulmonary disease (COPD) and history of pneumonia, respectively, are physician-confirmed. Bolded descriptors reached significance in terms of regression coefficients and 95% jack-knifed confidence intervals (ordered by strength of association to lung cancer, see Supplementary Fig. S3).

*Indicates variables that had an average regression coefficient with an inverse association to lung cancer (n = 28).

### OPLS models and performance

**Figure 3:**
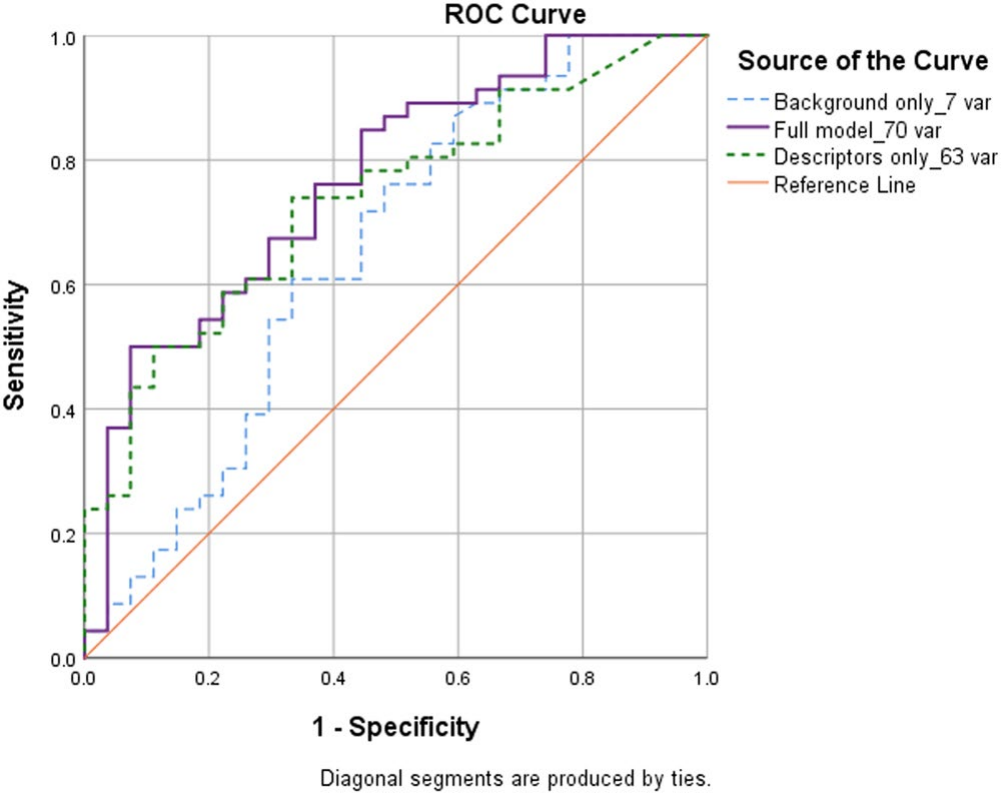
Receiver operating characteristic (ROC) curves for lung cancer prediction performance from orthogonal projections to latent structures (OPLS) modelling. ROC curves of lung cancer prediction performance were calculated from CV test set lung cancer prediction scores compared to diagnostic outcome (lung cancer or no cancer). Area under the ROC curves are shown in Table 3. For a detailed description of the full model and included variables, see Table 2. Background only_7 var (blue broken line): Seven background variables only. Full model_70 var (purple line): Final model, including 63 descriptors + seven background variables. Descriptors only_63 var (green broken line): 63 descriptors only.

The 145 descriptors were first modeled in OPLS together with the 16 background variables, which confirmed low contributions of the nine background variables removed in the PCA (OPLS VIP values < 1). The next model thus included 145 descriptors and seven background variables as in the final PCA. Thereafter, a trimmed OPLS model with 70 variables was discovered through an iterative optimisation process evaluating both maximal explained LC variance as well as best prediction of LC in the CV test set (AUC > 0.7) (Table 3). In brief, the model was trimmed by sequential removal of descriptors with no model contribution (Final Model: Fig. 2; excluded variables: Supplementary Table S2). Of relevancy for this study, the largest Youden’s index for sensitivity (0.402) was selected: sensitivity = 84.8%; specificity = 55.6%. Figure 3 illustrates the ROC curves for the final model, indicating diagnostic model performance from predicted scores from the CV test set, including the full model with 70 variables, the 63 descriptors only, or the seven background variables only. Fig. S2A,B demonstrates the final model selection of 63/145 descriptors with seven background variables through variable count vs. explained variance. The majority of selected descriptors were from the Breathing, Cough, and Pain/Aches/Discomfort modules (>8 from each, respectively) (Table 2).

**Table 3:** Lung cancer prediction performance from orthogonal projections to latent structures (OPLS).

Model	AUC	AUC2	C	R^2^X	R^2^	Q^2^	Sens	Spec
Full model, 70 variables	0.767	0.695	2	42.3	62.4	58.1	84.8*	55.6*
Descriptors only, 63 variables	0.736	0.670	2	32.7	56.0	50.1	73.9	66.7
Background only, 7 variables	0.652	0.568	2	79.9	51.7	50.9	76.1	51.9

Table headings: **AUC**: Area under the receiver operating characteristic (ROC) curve, cross-validation (CV) test set; **AUC2**: AUC, training set; **C**: Number of orthogonal components; **R**^2^**X**: Percent explained X variance (for all independent variables); **R**^2^: Percent explained Y variance (lung cancer); **Q**^2^: Cross-validated R^2^ (CV test set); **Sens/Spec**: Percent sensitivity and specificity, respectively, of the model in the CV test set, based off the optimal cutoff from the Youden’s index.

Model abbreviations: Full model: Final model with 70 variables (63 descriptors and seven background variables), built on maximal explained variance (R^2^ and Q^2^). After initially projecting all 145 descriptors (symptoms/sensations), candidates were then chosen in OPLS by visual inspection of regression coefficients and variable importance for the projection (VIP) values, with sequential removal of descriptors with no model contribution (S1 Table). The seven background variables were selected after demonstrating principal component analysis loadings > 0.1 and OPLS VIP values > 1. A full list of the final 70 variables is shown in Table 2. All sensitivity/specificity values are selected from the cutoff with the largest Youden’s index. Sensitivity was preferred in this study.

*Maximum performance of this model was with Youden’s index = 0.426 favoring specificity: sensitivity = 50%, specificity = 92.6%. Of relevancy for this study, the largest Youden’s index tailored for sensitivity (0.402) was selected: sensitivity = 84.8%; specificity = 55.6%.

All 70 variables were instrumental in maximal variance explanation and accurate LC prediction. However, should the prediction need to be centralised to one component, 14/42 positive predictors of LC were significantly predictive of LC (significant descriptors bolded in Table 2; all regression coefficients: Supplementary Fig. S3), which includes, in order of magnitude, background predictors: current smoking, cold/flu/pneumonia within the past two years, female sex, and older age; and the following descriptors: a cough that varied over the day, back pain/aches/discomfort, early satiety, appetite loss, having less strength, breathing worse upon exertion, haemoptysis/hematemesis, a heightened sensitivity to different smells, consistent aches, and a voice that got more rough/coarse. Of 28 LC-negatively-associated variables, having had antibiotics within the past two years had a significantly lower association to LC (Table 2; Supplementary Fig. S3).

**Figure 4:**
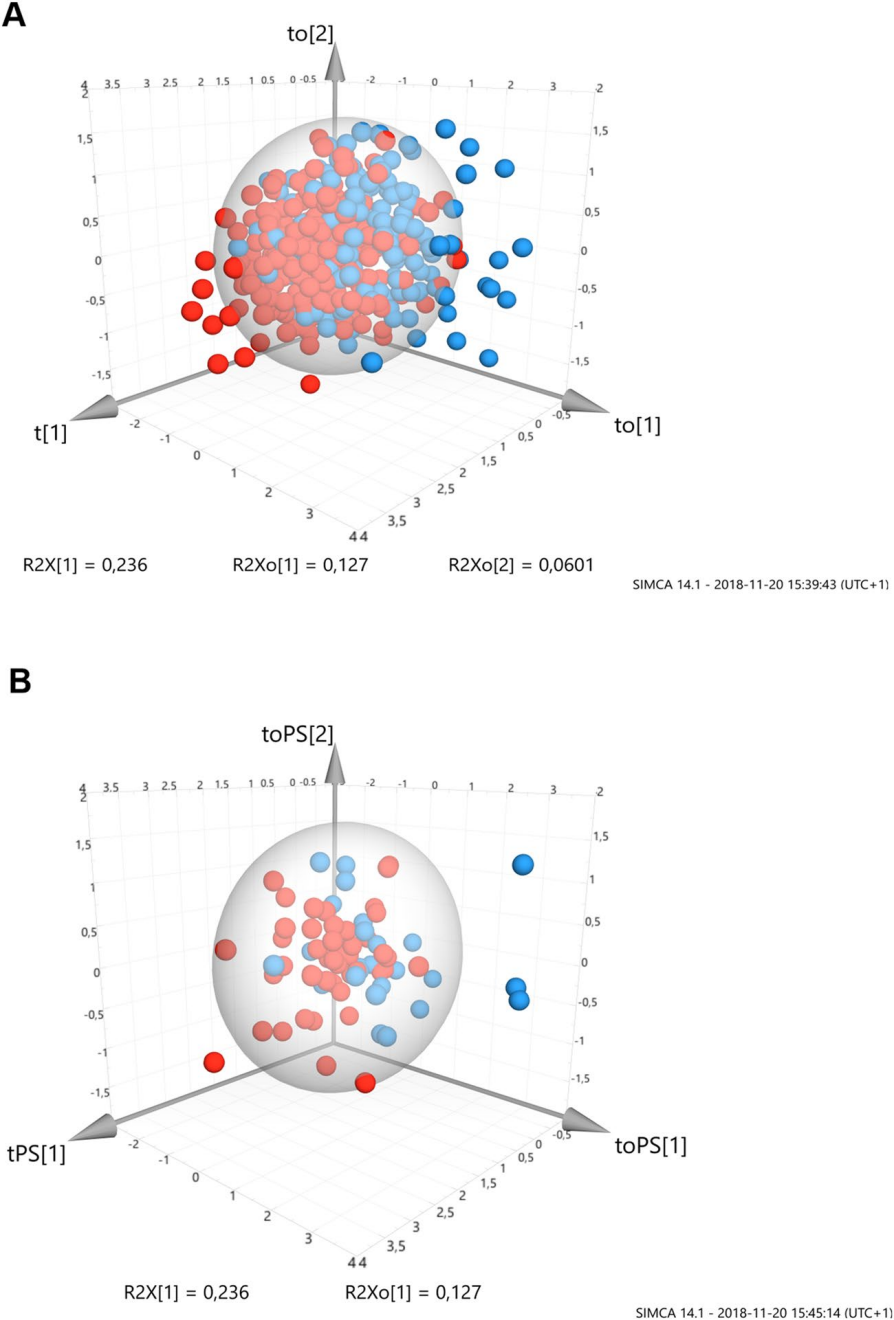
Orthogonal projections to latent structures (OPLS) 3D scores plot. Individual scores for the training set (**A** n = 433) and predicted scores (PS) for the cross-validated test set (**B** n = 73) are shown for the final model. All three of the OPLS model components are plotted, including the predictive component (t[1]) and the two orthogonal components (to[1] & [2]) (total R^2^X variance = 42.3%: t[1] = 23.6%, to[1] = 12.7%, to[2] = 6%). Predictive explained R^2^Y variance (lung cancer: training set): 62.4%; cross-validated explained Q^2^ variance (lung cancer: cross-validated test set): 58.1%. A total of 63 descriptors of symptoms and sensations were included together with seven background variables (Table 2). Coloured circles indicate lung cancer (red) or no cancer (blue). Outliers are indicated beyond the 95% confidence interval ellipse.

The 70-variable model resulted in accurate model performance in the CV test set (n = 73): area under the ROC curve = 0.767 (descriptors only: 0.736/background predictors only: 0.652), sensitivity = 84.8% (73.9/76.1%, respectively), specificity = 55.6% (66.7/51.9%, respectively). As indicated in the performance parameters, the seven background predictors alone (AUC = 0.652) failed to meet good diagnostic accuracy, while, upon excluding background predictors, independent LC prediction among descriptors was still demonstrated (AUC = 0.736) (Table 3). OPLS scores plots and all three components for the final model training set and CV test set are shown in Fig. 4A,B, respectively, and a biplot with both scores and variable loadings in Supplementary Fig. S4.

## Discussion

To our knowledge, this is the first study to utilise an interactive e-questionnaire given to individuals referred for LC investigation to comprehensively analyse and identify pre-diagnostic descriptors of symptoms and sensations related to LC. The unique, individualised e-questionnaire that we utilised had a design that allowed us to cover a large number of questions while minimising patient burden. Furthermore, this was combined with a cutting-edge multivariate machine learning analysis of multi-dimensional data to probe how combinations of variables perform in predicting LC. Given the highly variable and heterogeneous symptoms and sensations which were reported, OPLS regression was essential for analysis due to its filtration capability in capturing and centralising predictive variation despite the complexity of our data.

Several cohort risk prediction studies that analysed diffuse general practice medical records^17–20^ and a limited survey^22^ previously identified haemoptysis, dyspnoea, chest pain, cough, weight loss, appetite loss, voice hoarseness, and/or fatigue up to two years before diagnosis as LC risk signs. A recent systematic literature review and meta-analysis highlighted haemoptysis, dyspnoea, cough, and chest pain to be key contributors^30^. Our results are in line with most of these previously-reported early risk factors, including haemoptysis, dyspnoea (breathing worse upon exertion), cough problems (cough that varied over the day), appetite loss, and voice hoarseness; and – in addition to active smoking as the most established risk factor – COPD^18,19^ and relatively recent lower/upper respiratory or non-specific chest infections^19^. On the other hand, through our investigation we identified a plethora of new, early, pre-diagnostic descriptors derived from the patient experience, i.e. early satiety; back pain/aches/discomfort (which could either imply lower or upper back pain; previous models specifically reported only chest pain); having less strength; a heightened sensitivity to different smells; and consistent aches. The identification of these unique descriptors was enabled through the use of an individualised e-questionnaire based on inductive research systematising patients’ experiences.

Regarding other risk factors, female sex predicts LC in our results from a Swedish urban setting, which is a disturbing finding. The trend over the past several decades with more women smoking in Sweden points to a need for more cessation programs for women^31^. Finally, we could not confirm that the following previously-reported independent risk signs were predictive of LC, primarily due to exclusion from investigation due to lack of observations or not investigating the phenomena, or from a lack of model contribution: thrombocytosis or abnormal spirometry^17^, socioeconomic status^18,19^ or family history of cancer (not investigated, respectively)^18^; other/prior cancer (our endpoint was primary LC only and including this could overfit the model)^18^; and finger clubbing (nail changes)^17^, anaemia^18^ or a chronic cough with chronic phlegm (removed due to lack of model contribution)^32^. We did have information on self-reported weight and weight loss, however, this was missing in a large proportion of patients and we therefore could not draw conclusions other than to state we saw a trend that confirms their inclusion as valuable potential LC predictors as has been previously demonstrated^18,19^.

Two large aforementioned cohort studies have thus far created cross-validated models that include early symptoms with diagnostic performance from patient medical records denoting potential LC risk signs up to two years prior to diagnosis^18,19^. The first model^18^, with haemoptysis, dyspnoea, cough, and appetite loss, had a mean 72% cross-validated explained variation, 0.92 AUC, and 77.3% sensitivity for a top 10% risk score (specificity not reported) (additional background variables included body mass index and weight loss, lower socioeconomic status, ordinal smoking status (cigarettes/day), and, among females, prior cancer). The second model^19^, with haemoptysis, dyspnoea, chest pain, cough, and voice hoarseness, had a 0.88 AUC and a peak sensitivity of 93.98% vs. 59.67% specificity in cross-validation (explained variance not reported) (additional background variables included lower socioeconomic status, weight loss, and smoking history (current, past or ordinal by cigarettes/day)). These metrics can be compared with the performance of our model, with cross-validated explained variance of 58.1%; AUC: 0.767, and 84.8% peak sensitivity vs. 55.6% specificity. While these studies have major strengths in their nationally-representative sample sizes and AUC metrics that outperform our model, they have methodological limitations addressed in our study. In both prior studies, comorbid/previous cancers other than LC were not excluded, leading to a very heterogeneous sample with findings less clinically relevant to primary LC only, in relation to no cancer at all. Additionally, their data derives from general practice record retrieval of a limited set of diffuse symptoms (i.e. cough, chest pain, and dyspnoea), and quality control of descriptors was not possible due to the lack of direct patient interaction. Our findings are thus both robust and novel as we know of no other study using detailed patient-reported descriptors of symptoms and sensations to predict primary LC.

This study has some limitations to consider, including potential patient recall bias due to the retrospective approach. Secondly, predictors could have been made more precise, such as including pack years as opposed to using only current smoking status. Additionally, the predictive value of several rarely-occurring early descriptors could not be determined in our study. Therefore, a larger sample would help in finding the potential importance of these descriptors. With this in mind, while our model accurately predicted LC among a population of at-risk patients who already passed general practice gatekeepers and were subsequently referred to lung specialists, our model also needs to be tested against a more general population to determine its validity as a potential tool to help flag patients early for diagnostic workup.

The present study was able to identify unique early patient-reported descriptors predictive of LC among a vast array of 285 descriptors investigated through an advanced modelling approach from data collected with an interactive tablet questionnaire tailored for usability. While several LC descriptors identified by us have been previously described, our unique approach allowed identification of novel descriptive indicators of LC risk that can be integrated into a simplified questionnaire in future LC investigation. Signs of early satiety before diagnosis and treatment, for example, was a major early LC predictor in the current study that has, to our knowledge, not been identified before. Our specific, in-depth and complex investigation allowed for key descriptors to surface, and such an approach requires an advanced method like OPLS to handle the magnitude of variables by projection instead of being directly influenced by- or needing to control for the amount of variables^23–26^. As a potential tool for use in clinical practice, the 70 variables identified may at a later stage be administered as a questionnaire to individuals exhibiting respiratory-related distress, whereby the resulting OPLS risk-prediction score may be used to flag patients for specialized diagnostic workup. Furthermore, PEX-LC could be tested to tackle the large false positive rate problem in conjunction with CT-based LC screening to prioritize patient selection from large risk-group populations.

## Conclusions

This is a first step towards identifying optimal patient-reported predictive markers for LC, and combining these with relevant biological markers may represent the most promising means to reduce LC mortality apart from smoking cessation. The results from this advanced modelling approach applied on early symptoms and sensations derived from an interactive e-questionnaire may lead to a tool for referral and LC diagnostic decision-making, thus potentially facilitating a more timely diagnosis and improving LC survival.

## Supplementary information

Supplementary Information

## Data Availability

Data cannot be shared publicly due to protecting the privacy of the patients who agreed to participate in the study. The anonymised dataset utilised for analyses carried out for the current study is available from the corresponding author on reasonable request.
