# Supplementary material for: Early symptoms and sensations as predictors of lung cancer: a machine learning multivariate model
Source: Sci Rep. 2019 Nov 11;9:16504. doi: 10.1038/s41598-019-52915-x (PMC6848139; doi:10.1038/s41598-019-52915-x)
Supplement: Supplementary file 1 — Supplementary Information [file 41598_2019_52915_MOESM1_ESM.docx]

**Early symptoms and sensations as predictors of lung cancer: a machine learning multivariate model**

Adrian Levitsky^1,2^, PhD; Maria Pernemalm^2^, PhD; Britt-Marie Bernhardson^1^, RN PhD;
Jenny Forshed^2^, MD PhD; Karl Kölbeck^3^, MD; Maria Olin^3^, RN MSc, Roger Henriksson^4^, MD PhD; Janne Lehtiö^2^, PhD; Carol Tishelman^1,5,6^, RN PhD; Lars E. Eriksson^1,7,8^, RN PhD*

^1^ Division of Innovative Care Research, Department of Learning, Informatics, Management and Ethics (LIME), Karolinska Institutet, SE-171 77 Solna, Sweden

^2^ Cancer Proteomics Mass Spectrometry, Department of Oncology-Pathology, Karolinska Institutet, Science for Life Laboratory, SE-171 65 Solna, Sweden

^3^ Lung Oncology Center, Cancer Theme, Karolinska University Hospital, SE-171 76 Solna, Sweden

^4^ Department of Radiation Sciences and Oncology, University of Umeå, SE-901 87 Umeå, Sweden

^5^ Center for Health Economy, Informatics and Health System Research (CHIS), Stockholm Health Care Services (SLSO), Stockholm County Council, SE-113 65 Stockholm, Sweden

^6^ The Center for Rural Medicine (Glesbygdsmedicinskt Centrum GMC), Västerbotten County Council, SE-923 31 Storuman, Sweden

^7^ School of Health Sciences, City, University of London, Northampton Square, London EC1V 0HB, United Kingdom

^8^ Department of Infectious Diseases, Karolinska University Hospital, SE-141 86 Huddinge, Sweden

* Corresponding Author: Lars E. Eriksson, Division of Innovative Care Research, Department of Learning, Informatics, Management and Ethics, Karolinska Institutet, SE-171 77 Solna, Sweden. E-mail: [lars.eriksson@ki.se](mailto:lars.eriksson@ki.se). Tel. +46 (0)8 524 000 00


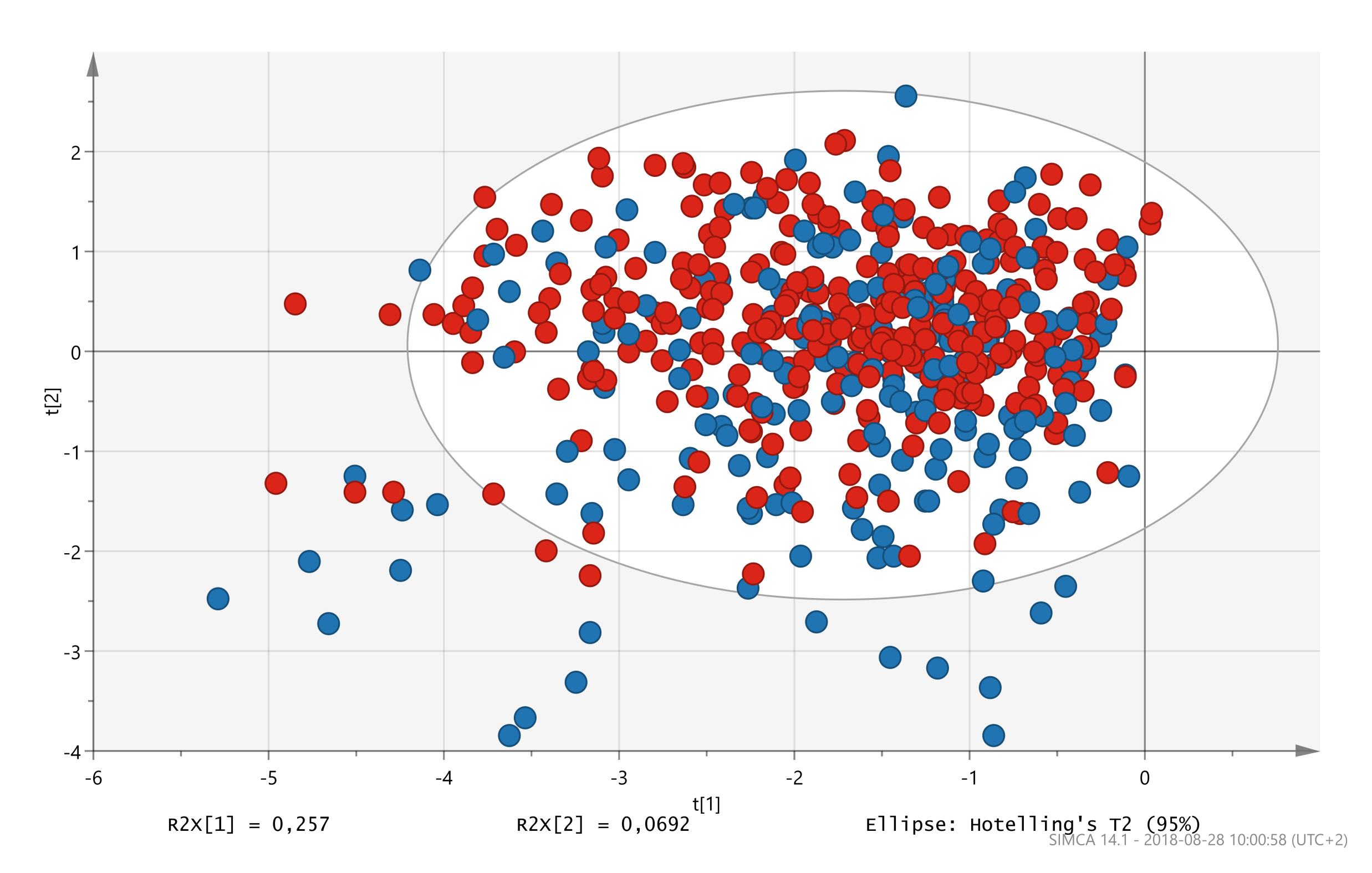


**Supplementary Fig. S1. Principal component analysis (PCA) scores plot of entire dataset.**

Individual scores for all participants (n=506) upon inclusion of all independent variables (X) (n=152) are shown in a two-component (t[1-2]) PCA model (explained R^2^X variance=32.61%: t[1]=25.7%, t[2]=6.91%). Seven background variables (variable importance for the projection values, VIP>1) were included (age, current smoking, sex, a physician-confirmed history of chronic obstructive pulmonary disease or pneumonia, respectively, or a cold/flu/pneumonia or antibiotics within the past two years, respectively) together with 145 descriptors. Colored circles indicate lung cancer (red) or no cancer (blue). Outliers are indicated beyond the 95% confidence interval ellipse.


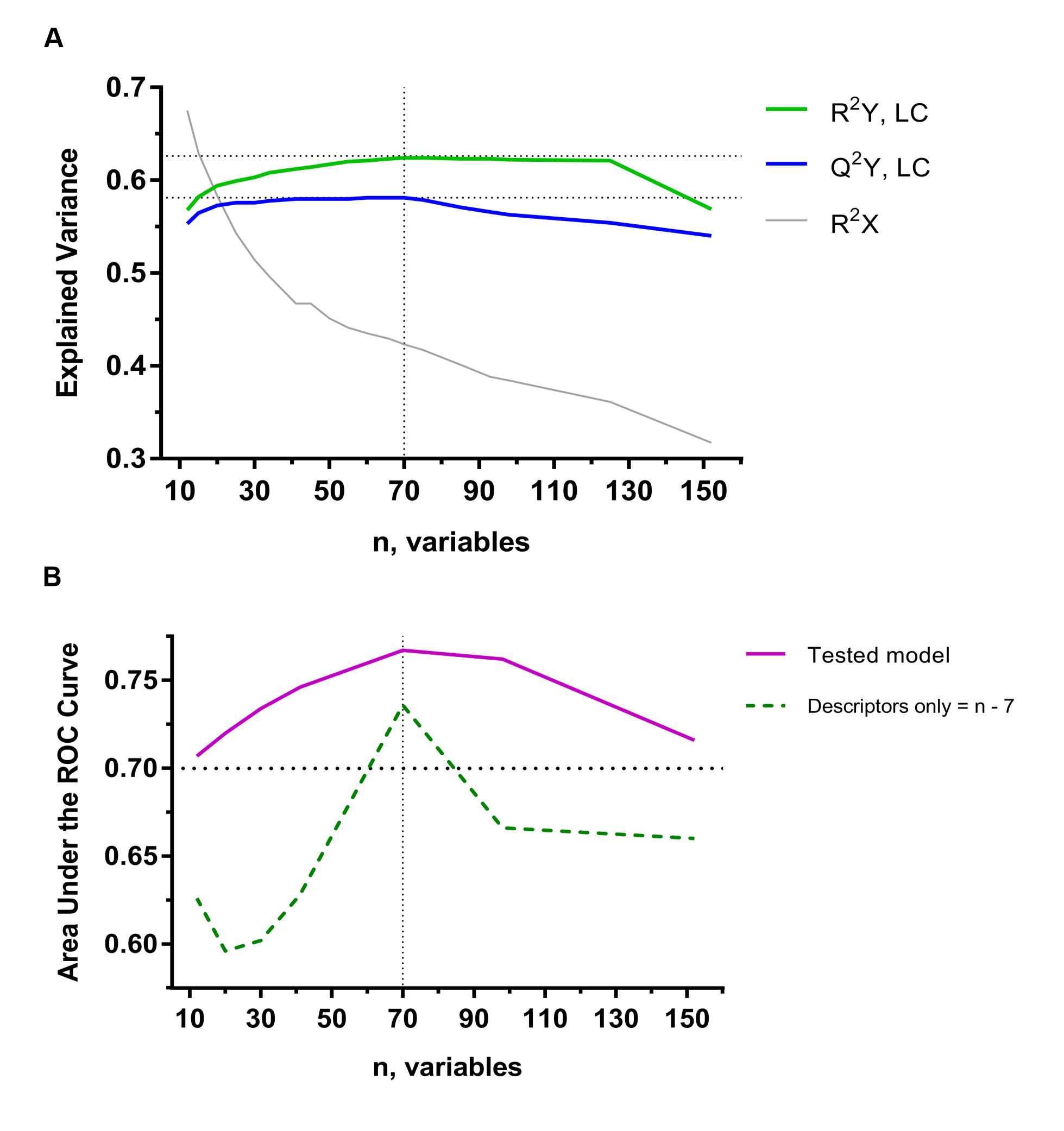


**Supplementary Fig. S2. Orthogonal projections to latent structures (OPLS) model performance vs. included variables.**

Indicators of model performance vs. variables are shown, with final selection of the 70-variable model indicated (dotted line). **A**: explained R^2^Y variance (lung cancer, LC, green line): 62.4%; cross-validated explained Q^2^ variance (LC: cross-validated test set, blue line): 58.1%; R^2^X variance (independent variables, grey line): 42.3%. **B**: Area under the receiver operating characteristic (ROC) curve values for each tested model by variable number (purple line). Exclusion of the seven background variables (green broken line) are also indicated (descriptors only = n-7).

In the final model, a total of 63 descriptors of symptoms and sensations were included together with seven background variables (Table 2).


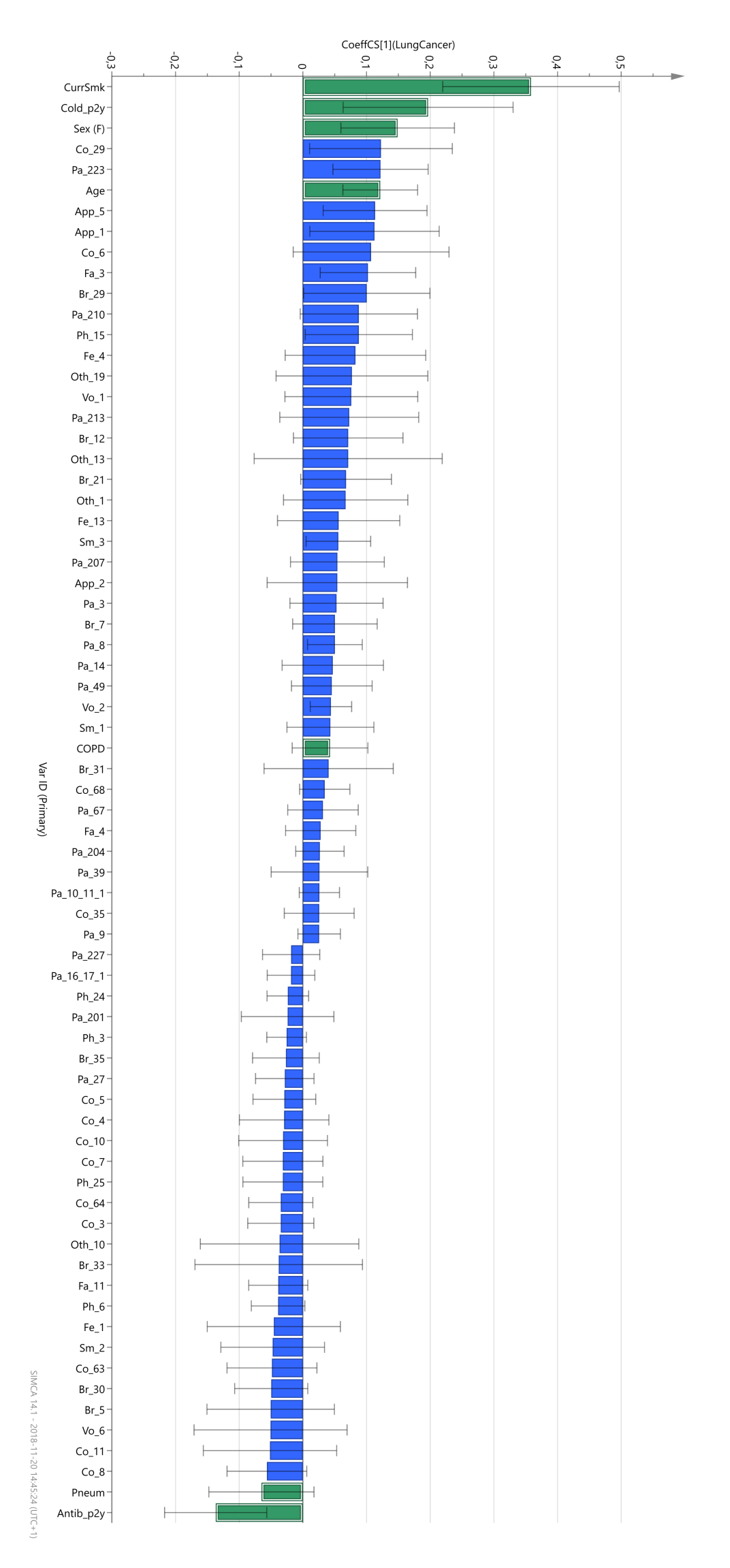


**Supplementary Fig. S3. Orthogonal projections to latent structures (OPLS) regression coefficients.**

Regression coefficients are shown for the full model with 70 variables. The coefficients represent the change in lung cancer when each variable varies from 0 to 1 (or +1 standard deviation for age: unit-variance scaled), while the other variables are kept at their averages. These 70 variables were attained after having originally inspected regression coefficients and variable importance for the projection (VIP) values among all 152 variables and removing those without variance contribution. Error bars indicate jack-knifed confidence intervals from cross-validation.

The seven background variables (colored green) include age, female sex (Sex (F)), current smoking (CurrSmk), physician-confirmed history of chronic obstructive pulmonary disease (COPD), a cold, flu, or pneumonia within the past two years (Cold_p2y), having had antibiotics within the past two years (Antib_p2y), and a physician-confirmed history of pneumonia (Pneum). For a detailed list of the 63 descriptors matching module+descriptor number, see Table 2. Module abbreviations: Br: Breathing, Co: Cough, Ph: Phlegm/Expectorates, Pa: Pain/Aches/Discomfort; Fa: Fatigue, Vo: Voice, App: Appetite/Taste/Eating; Sm: Olfactory (Smell); Fe: Fever; Oth: Other.


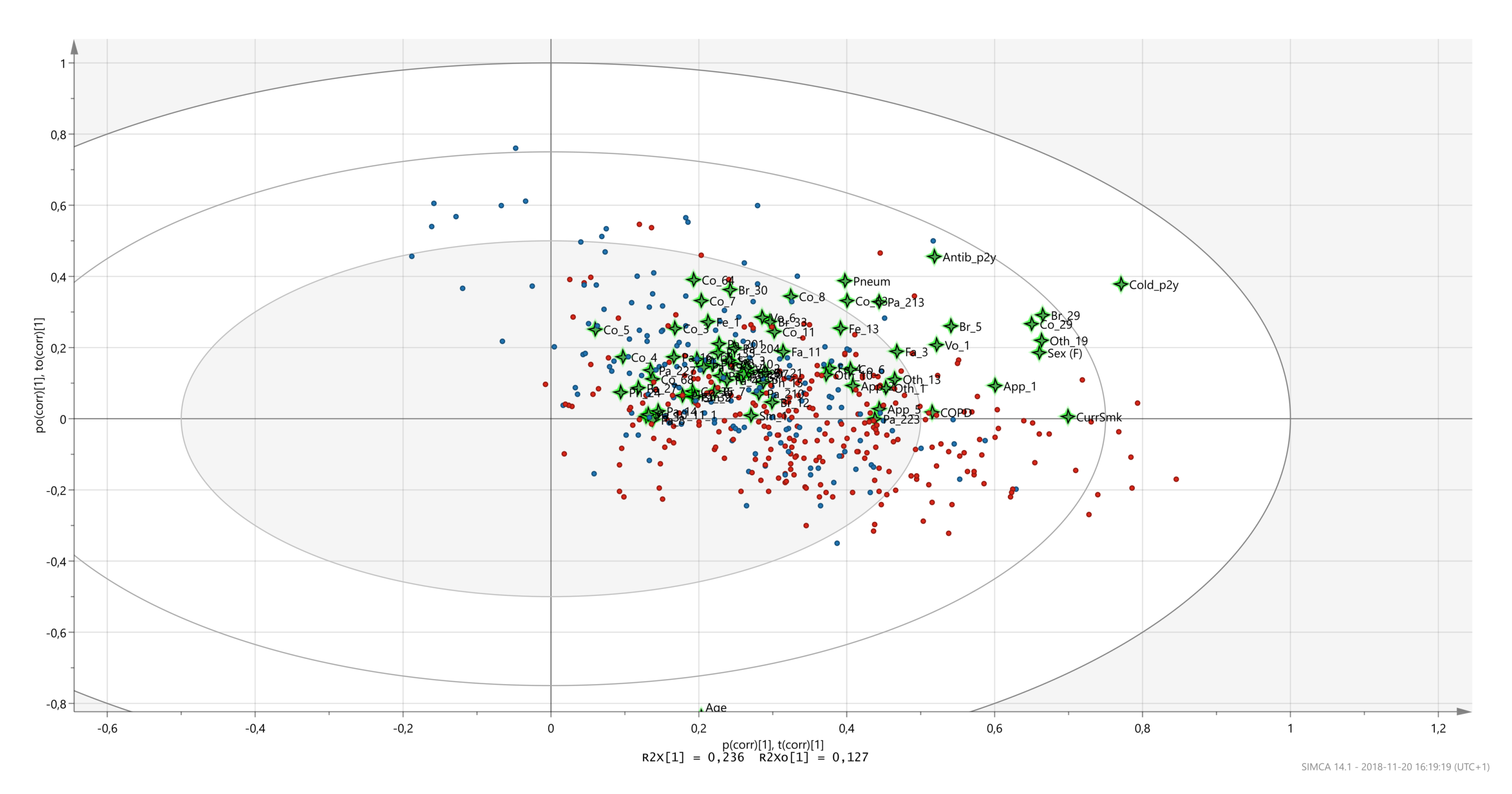


**Supplementary Fig. S4. Orthogonal projections to latent structures (OPLS) biplot.**

Individual scores for the training set (n=433) are shown together with loadings in a biplot for the final model. The first two of the OPLS model components are plotted (for all three components, see Fig. 4). Loadings and scores have been scaled as correlations using a scaling factor based on a ratio of the sum of squares of the loadings to the sum of squares of the scores. The three correlation circles range from smallest to largest: correlations of 0.5, 0.75, and 1.0, respectively (correlations below 0.5 are filled in grey). Variables that load to the right side of the x-axis indicate a stronger correlation to lung cancer (component 1: predictive), whereas high loadings on the second component (y-axis) correlate more strongly to orthogonal variation.

A total of 63 descriptors of symptoms and sensations were included together with seven background variables (Table 2). Colored circles indicate lung cancer diagnosis (red) or no cancer (blue). Green stars indicate loadings of the 70 variables in the model (Table 2).

**Supplementary Table S1. First exclusion of descriptors and background items by module, primarily due to limited observations.**

| Background | |
| --- | --- |
| Living with a husband/wife/partner | Weight, 3 months prior |
| Living with another adult (yes/no) | Weight, 6 months prior |
| Living with another adult, who? (e.g. mother, father) | Weight, 1 year prior |
| Living with young children | Smoking status: Other (specify) |
| Living alone | Years smoked |
| Highest finished level of education (e.g. university) | Changed smoking habits, past two years (more/less/other/no) |
| Highest finished level of education: other (specify) | Changed smoking habits, past two years (specify) |
| Native country | Time since smoking cessation (months) |
| Working full-time | Quit smoking due to breathing problems/other chest problems |
| Working part-time | Quit smoking due to cough |
| Unemployed | Quit smoking: not feeling well from smoking (yes/no; specify) |
| Studying | Quit smoking due to recommendation by medical staff |
| Retired | Quit smoking for relatives’ or others’ sake |
| On sick-leave | Quit smoking through relatives’ initiative |
| Other working situation | Quit smoking through relatives’ health as a warning sign |
| Other physician-reported comorbidity (yes/no) | Quit smoking for financial reasons/smoking costs too much |
| Other physician-reported comorbidity (specify) | Quit smoking due to smelling bad from smoking |
| No physician-reported comorbidities | Quit smoking due to no good reason to smoke/why smoke? |
| Weight change, past year (no/unknown/weight loss/gain) | Quit smoking due to other reason (yes/no) |
| Current weight (kg) | Quit smoking due to other reason (specify) |
| Weight, 1 month prior |  |
|  | |
| Breathing | |
| Other breathing problems^a^ | Breathing problems worsened during the evening |
| Breathing sound: Other^a^ | Breathing problems worsened during the night |
| Breathing sound: None of the above^a^ | Breathing problems worsened due to other reason^a^ |
| Breathing problems varied over the day | Breathing problems improved due to other reason^a^ |
| Breathing problems worsened during the morning | Breathing problems not influenced by any of the above^a^ |
| Breathing problems worsened during daytime |  |
|  | |
| Cough | |
| Not like any other kind of cough | Cough worsened during daytime |
| Other type of cough^a^ | Cough worsened during the evening |
| Cough location: Throat | Cough worsened during the night |
| Cough location: Trachea | Worsened cough was different for different days |
| Cough location: Chest | Cough worsened during winter |
| Cough location: Upper chest | Cough worsened during spring |
| Cough location: Behind the ribcage | Cough worsened during summer |
| Cough location: Against the back | Cough worsened during fall |
| Cough location: On the right side | Cough worsened during pollen season |
| Cough location: On the left side | Worsened cough varied from year to year |
| Cough location: Difficult to say, not certain | Cough got better from the cold (temperature/weather) |
| Cough location: Other location^a^ | Cough worsened due to other reason^a^ |
| Cough location: Don’t know^a^ | Cough improved due to other reason^a^ |
| Cough worsened during the morning | Cough not influenced by any of the above^a^ |
|  | |
| Phlegm/Expectorates | |
| Coloured phlegm/expectorates | Other consistency in phlegm/expectorates^a^ |
| Green phlegm/expectorates* | Changes in phlegm/expectorates: Don’t know^a^ |
| Yellow phlegm/expectorates* | Phlegm/expectorates worsened during the morning |
| Red phlegm/expectorates* | Phlegm/expectorates worsened during the daytime |
| Brown phlegm/expectorates* | Phlegm/expectorates worsened during the evening |
| Cloudy/murky/unclear phlegm/expectorates* | Phlegm/expectorates worsened during the night |
| Other colour change of phlegm/expectorates* | Phlegm/expectorates: same the whole day |
| Blood-mixed phlegm/expectorates* | Phlegm/expectorates: different for different days |
| Blood-mixed phlegm/expectorates with a clear-red colour* | Phlegm/expectorates worsened during other time^a^ |
| Blood-mixed phlegm/expectorates with a dark colour* | Most problematic time of phlegm/expectorates: Don’t know^a^ |
| Blood-mixed phlegm/expectorates with another appearance* |  |
|  | |
| Pain/Aches/Discomfort | |
| Hurting** | Tenderness: Worsens when changing body position |
| Hurting: Continues/worsens when breathing* | Pressure sensation** |
| Hurting: Reduces/improves when changing body position* | Pressure sensation: Continues/worsens when breathing* |
| Hurting: Persists/worsens when changing body position* | Pressure sensation: Improves when changing body position* |
| Aches** | Pressure sensation: Worsens when changing body position* |
| Aches: Continues/worsens when breathing* | Clump/swelling or feeling of an obstruction** |
| Aches: Reduces/improves when changing body position* | Clump/swelling: Continues/worsens when breathing* |
| Aches: Persists/worsens when changing body position* | Clump/swelling: Improves when changing body position* |
| Pain** | Clump/swelling: Worsens when changing body position* |
| Pain: Continues/worsens when breathing* | Heartburn: Consistent |
| Pain: Reduces/improves when changing body position* | Heartburn: Comes and goes |
| Pain: Persists/worsens when changing body position* | Heartburn: Continues/worsens when breathing |
| Burning pain/aches: Consistent | Heartburn: Improves when changing body position |
| Burning pain/aches: Comes and goes | Heartburn: Worsens when changing body position |
| Burning pain/aches: Continues/worsens when breathing | Feeling of uneasiness that is difficult to describe** |
| Burning pain/aches: Improves when changing body position | Feeling of uneasiness: Continues/worsens when breathing* |
| Burning pain/aches: Worsens when changing body position | Feeling of uneasiness: Improves when changing body position* |
| Cramping pain/aches** | Feeling of uneasiness: Worsens when changing body position* |
| Cramping pain/aches: Consistent | Other pain/aches/discomfort problems^a^ |
| Cramping pain/aches: Continues/worsens when breathing* | Pain/aches/discomfort: Throat, right side |
| Cramping pain/aches: Improves when changing body position* | Pain/aches/discomfort: Throat, left side |
| Cramping pain/aches: Worsens when changing body position* | Pain/aches/discomfort: Shoulder blade, right side |
| Stabbing pain/aches** | Pain/aches/discomfort: Shoulder blade, left side |
| Stabbing pain/aches: Improves when changing body position | Pain/aches/discomfort: Shoulders, right side |
| Stabbing pain/aches: Worsens when changing body position | Pain/aches/discomfort: Shoulders, left side |
| Dull pain/aches** | Pain/aches/discomfort: Neck, right side |
| Dull pain/aches: Continues/worsens when breathing* | Pain/aches/discomfort: Neck, left side |
| Dull pain/aches: Improves when changing body position* | Pain/aches/discomfort: Chest, high up |
| Dull pain/aches: Worsens when changing body position* | Pain/aches/discomfort: Chest, behind the ribcage |
| Sticking pain/aches** | Pain/aches/discomfort: Chest, against the back |
| Sticking pain/aches: Continues/worsens when breathing* | Pain/aches/discomfort: Chest, right side |
| Sticking pain/aches: Improves when changing body position* | Pain/aches/discomfort: Chest, left side |
| Sticking pain/aches: Worsens when changing body position* | Pain/aches/discomfort: Location, hard to say/don’t know |
| Tenderness: Consistent | Pain/aches/discomfort: Back, right side |
| Tenderness: Comes and goes | Pain/aches/discomfort: Back, left side |
| Tenderness: Continues/worsens when breathing | Pain/aches/discomfort: Other place^a^ |
| Tenderness: Improves when changing body position | No pain/aches/discomfort in a specific bodily location^a^ |
|  | |
| Fatigue | |
| Other problems with fatigue^a^ |  |
|  | |
| Voice Changes | **Appetite/Taste/Eating Changes** |
| Voice changes that are difficult to describe^a^ | Appetite/taste/eating changes that are difficult to describe^a^ |
| Other voice changes^a^ | Other appetite/taste/eating changes^a^ |
|  | |
| Olfactory Changes | **Fever** |
| Olfactory changes that are difficult to describe^a^ | Other fever changes^a^ |
| Other olfactory changes^a^ |  |
|  | |
| Other | |
| Other changes in temperament* |  |
| A feeling of uneasiness/feeling as if something is wrong* |  |
| Other changes* |  |
|  | |

Descriptors that did not meet inclusion criteria (at least four observations with answers of “yes” for lung cancer and no cancer, respectively) or were excluded for another reason (described below) are shown (n=140), in addition to excluded background variables (n=41). Additional reasons for exclusion included lack of univariate association to lung cancer.

Bolded descriptors were significant univariate associates of lung cancer, however, were not included due to the potential risk of overfitting the model due to being tightly linked with other known predictors (current smoking).

**^a^** Indicates descriptors that were unspecific and thus excluded, e.g. “other”, “none of the above”, or “don’t know”

* Indicates variables that were excluded individually but were merged together (and included in the analysis in a single variable for each phenomena, respectively) due to limited observations and/or sharing similar information. The following variables were created: Green/yellow/cloudy/other phlegm/expectorates; Haemoptysis/hematemesis (blood-mixed/red/brown/black phlegm/expectorates); Positional/breathing-based Pain, Aches, Hurting, Cramping pain/aches, Dull pain/aches, Sticking pain/aches, Pressure sensation, Clump/swelling, and a Feeling of uneasiness, respectively; and Other uneasiness and/or a feeling something is wrong.

** Indicates variables on a higher, less in-depth data level, and were not analysed in favor of more specific information.

Weight information was only available for individuals who reported they had a weight change within the past year, and, therefore, weight was not available for the entire sample and thus not included in the analysis.

An additional 10 variables were a quality check-up of filling in each respective module where the individual would answer “no” to problems (e.g. no to any breathing problems, any cough problems, etc.). Since these were not symptoms descriptors, they are not included above, but were nonetheless modelled in a sensitivity analysis and no accurate predictive model could be produced (results not shown).

There were additional variables not included due to an ordinal structure and were recoded to binary to suit the structure of the analysis. These included cold/flu/pneumonia within the past 2 years (0; 1-2; 3-5; >5 times), antibiotics within the past 2 years (0; 1-3; >3 prescriptions), and ordinal smoking status (never, other, past, current).

**Supplementary Table S2. Second exclusion of descriptors and background items by module, primarily due to lack of model contribution.**

| Background | |
| --- | --- |
| Past smokers, quit >1 year ago (vs. non-smokers) | Confirmed history of chronic bronchitis |
| Confirmed history of emphysema | Confirmed history of anaemia |
| Confirmed history of asbestos-related disease | Confirmed history of fluid in lungs (pulmonary oedema) |
| Confirmed history of heart (cardiovascular) disease | Confirmed history of angina pectoris |
| Confirmed history of asthma |  |
|  | |
| Breathing | |
| Hard to get air | Felt like a sore in the chest |
| Feeling pressure | Breathing sound: Squeaked, as if through a pipe |
| Hard to breathe deeply | Breathing sound: Rattled/wheezed |
| Laboured breathing | Breathing sound: Jarred, raspy |
| Hard to catch breath | Breathing sound: Bubbled, gurgled |
| Feeling of choking/panic | Breathing sound: Hissed |
| Feeling of uneasiness that is hard to describe | Breathing worsened when I spoke |
| Stabbing sensation from taking deep breaths | Breathing relieved by high humidity |
| Felt like a lump in the chest | Breathing relieved by coldness |
|  | |
| Cough | |
| Mucus cough | Cough worsened when I lay down |
| Dry cough | Cough got better when I lay down |
| Persistent cough | Cough woke me when I slept |
| Constant cough | Cough relieved by high humidity |
| Cough occurred/worsened when I spoke | Cough worsened by coldness |
|  | |
| Phlegm/Expectorates | |
| Increased amount | Lumps/pieces in sputum |
| Green/yellow/cloudy mucus or sputum | Thick consistency |
|  | |
| Pain/Aches/Discomfort | |
| Hurting: Consistent | Sensation of pressure: Consistent |
| Hurting: Positional/breathing-based | Sensation of pressure: Comes and goes |
| Pain: Comes and goes | Sensation of pressure: Positional/breathing-based |
| Burning aches/pain | Lump, swelling, or obstruction sensation: Consistent |
| Cramps: Positional/breathing-based | Lump, swelling, or obstruction sensation: Comes and goes |
| Stabbing aches/pain: Consistent | Lump/obstruction sensation: Positional/breathing-based |
| Stabbing aches/pain: Comes and goes | Feeling of discomfort/uneasiness, hard to describe: Consistent |
| Stabbing aches/pain: Continues/worsens when breathing | Feeling of discomfort/uneasiness: Comes and goes |
| Dull aches/pain: Consistent | Feeling of discomfort/uneasiness: Position/breathing-based |
| Dull aches/pain: Positional/breathing-based | Pain radiates between shoulder to chest |
| Sticking aches/pain: Consistent | Pain/aches/discomfort: Head |
| Sticking aches/pain: Comes and goes | Pain/aches/discomfort: Whole body |
| Sticking aches/pain: Positional/breathing-based |  |
|  | |
| Fatigue | |
| Less energy to do things | Felt worn out |
| Less will to do things | Felt abnormally/unhealthily tired |
| Difficult to stay awake | Felt out of sorts |
| Did not feel well-rested | Felt tiredness, weakness, or lack of energy that came and went |
| Increased need for sleep | Had a feeling of discomfort/uneasiness, hard to describe |
|  | |
| Voice Changes | **Appetite Changes** |
| Voice got weaker | Food/drinks tasted different |
| Lost my voice | Had trouble swallowing |
| Changed pitch, higher/lower |  |
|  | |
| Olfactory Changes | **Fever** |
| *No additional descriptors removed* | Fever |
|  | Day sweats: More than usual |
|  | Sweating all the time |
|  | Got cold feet |
|  | |
| Other | |
| Swollen/tender joints | Felt more down |
| Nail changes | More irritable |
| Felt thickness in throat | Other uneasiness and/or a feeling something is wrong |
|  | |

Excluded background variables (n=9) and descriptors (n=82) are shown. The first round of excluded descriptors (n=140) and background variables (n=41), primarily due to not meet inclusion criteria (at least four observations in each group, lung cancer or no cancer), are shown in Supplementary Table S1. Past smokers (bolded) as a variable was not included due to the potential risk of overfitting the model, as current smokers included those who quit smoking within the past 1 year. Background variables pertaining to history are physician-confirmed.
